# Ab initio determination on diffusion coefficient and viscosity of FeNi fluid under Earth’s core condition

**DOI:** 10.1038/s41598-022-24594-8

**Published:** 2022-12-08

**Authors:** Wei-Jie Li, Zi Li, Zhe Ma, Ping Zhang, Yong Lu, Cong Wang, Qian Jia, Xue-Bin Cheng, Han-Dong Hu

**Affiliations:** 1 Intelligent Science and Technology, Academy Limited of CASIC, Beijing, 100144 People’s Republic of China; 2 Institute of Applied Physics and Computational Mathematics, Beijing, 100088 People’s Republic of China; 3 Tianfu Innovation Energy Establishment, Chengdu, 610213 China; 4 Center for Applied Physics and Technology, Peking University, Beijing, 100871 People’s Republic of China; 5 College of Mathematics and Physics, Beijing University of Chemical Technology, Beijing, 100029 China; 6 X LAB, The Second Academy of CASIC, Beijing, 100854 People’s Republic of China

**Keywords:** Geochemistry, Geophysics

## Abstract

The Earth’s outer core is mainly composed of Fe and Ni. The geodynamo of the Earth’s core are closely correlated with the transport properties of the fluid in the Earth’s core. We selected the typical FeNi fluid, and systemically calculated its diffusion coefficient and viscosity under Earth’s core condition by quantum molecular dynamics simulation. The diffusion coefficients are almost constant along the core adiabatic curve. The self-diffusion coefficients of Ni along the core adiabatic curve range from 2.47 × 10^−9^ to 3.37 × 10^−9^ m^2^s^−1^. The diffusion coefficient increases with temperature increase, while viscosity decrease with temperature increase. The calculations on the transport properties suggest that the Ni impurities have a negligible effect on the diffusion coefficient and viscosity of Earth’s core.

## Introduction

Accurate knowledge of the physical properties under extreme pressure–temperature conditions is of considerable interest in various fields of physics, including the Earth system^1^, planetary physics^2^, astrophysics^3^, and inertial confinement fusion^4^. The center of the Earth consists of a solid inner core, surrounded by a spherical shell of the liquid outer core. The vigorous convection in the metallic liquid outer core thus powers the dynamo that sustains the magnetic field. The geodynamo is extremely sensitive to core conditions, which is a very active research topic in Earth science. The viscosity and diffusion coefficients of the Earth’s core are the main parameters in the convection process.

In the early reported paper^5^, the diffusion coefficient in the diffusion equation is mostly adopted as 3×10^−9^ m^2^s^−1^. It is difficult to get the accurate diffusion coefficient and viscosity values under Earth’s core condition experimentally^6^. Now, quantum molecular dynamics (QMD) can give a direct and quantitative estimation of the transport coefficients^7^. The QMD results show that the self-diffusion coefficient of Fe is 5.2×10^−9^ m^2^/s, and viscosity is 8.5 mPa s at T = 4300 K and density ρ = 10,700 kg m^−3^^8^. Recently, light element effects on transport properties were also considered, such as Fe–Si–O fluid^9^, Fe–O fluid^10,11^, Fe–S fluid^12^.

The QMD results of viscosity are far lower than the values inferred from seismic and other measurements^13,14^. Early in 1998, it was inferred that the viscosity of the inner core is $1.22 \times 10^{11}$ Pa s^15^. Geodynamic estimation by Buffett inferred viscosity is less than $10^{16}$ Pa s^16^. The theoretical value for viscosity varies largely by different methods, from ~ 1 mPa s^14,17^ under the outer condition to 10^11^ Pa s^15^ under the inner core condition. QMD calculation shows that the viscosity is 13 mPa s and diffusion coefficients $5 \times 10^{ - 9}$ m^2^s^−1^ at the inner-core boundary(ICB), and 12 mPa and $4 \times 10^{ - 9}$ m^2^s^−1^ at the core-mantle boundary (CMB)^18^. The viscosity is about several mPa s for MD results of FeNi fluid^19^. Though there were limitations in MD, the early reported MD results claim that Ni has a negligible effect on viscosity^17^. However, the MD results dependent on the empirical parameters of atom potential, and the self-diffusion coefficient of Fe and Ni are not fully reported. AIMD is an optimal method for the calculation of ionic transport properties without empirical parameters.

However, the self-diffusion coefficient and viscosity of Fe–Ni fluid at Earth’s core condition have never been calculated by QMD. In this paper, we calculated the diffusion coefficients and viscosity of Fe–Ni fluid under Earth's core condition by the precisely QMD methods, and give a simple analysis of the temperature effect on transport properties of Fe–Ni fluid.

## Methods and calculations

### Transport property

The theory equations are collected from Ref.^7,20–22^. Diffusion coefficients can be calculated by either velocity autocorrelation functions or mean-squared displacements by equilibrium molecular simulation. The self-diffusion coefficient *D*_*i*_ of element *i* is calculated by the Einstein equation from velocity correlation function *A(t)* is

1 $$ \begin{gathered} A\left( t \right) = \left\langle {{\mathbf{V}}_{i} \left( t \right){\mathbf{V}}_{i} \left( 0 \right)} \right\rangle , \hfill \\ D_{i} = \frac{1}{3}\int_{0}^{\infty } {A\left( \tau \right)d\tau } , \hfill \\ \end{gathered} $$

where *V*_*i*_*(t)* is the velocity of species *i* at time *t*. and < … > is an average of velocity correlation function over atoms. From the sight of mean square displacement (MSD) *M(t)*, *D*_*i*_ is the asymptotic slope of *M(t)* as a function of time:

2 $$ \begin{gathered} M\left( t \right) = \frac{1}{{N_{I} }}\sum\limits_{{N_{I} }} {\left\langle {\left| {{\mathbf{R}}_{i} \left( t \right) - {\mathbf{R}}_{i} \left( 0 \right)} \right|^{2} } \right\rangle } , \hfill \\ D_{i} = \mathop {\lim }\limits_{t \to \infty } \frac{M\left( t \right)}{{6t}}, \hfill \\ \end{gathered} $$

where *R*_*i*_*(t)* is the trajectory of a species atom *i*, and *N*_*I*_ is the number of ions.

Calculated by the Green–Kubo relation, the shear viscosity *η* is the integral of the autocorrelation function of the off-diagonal component of the stress tensor (ACF) *S(t)*.

3 $$ \begin{gathered} C_{\alpha \beta } \left( t \right) = \left\langle {P_{\alpha \beta } \left( {t + t_{0} } \right)P_{\alpha \beta } \left( {t_{0} } \right)} \right\rangle \hfill \\ \eta = \frac{V}{{k_{B} T}}\mathop {\lim }\limits_{t \to \infty } \int_{0}^{t} {S\left( {t^{\prime}} \right)dt^{\prime}} \hfill \\ S(t) = \frac{1}{5}\left\{ {C_{xy} \left( t \right) + C_{yz} \left( t \right) + C_{zx} \left( t \right) + \frac{1}{2}\left[ {C_{xx} \left( t \right) - C_{yy} \left( t \right)} \right] + \frac{1}{2}\left[ {C_{yy} \left( t \right) - C_{zz} \left( t \right)} \right]} \right\} \hfill \\ \end{gathered} $$

where *k*_*B*_ is the Boltzmann factor, *T* is temperature and *V* is the volume of the cell. The results are averaged from the five independent off-diagonal components of the stress tensor *P*_*xy*_, *P*_*yz*_, *P*_*zx*_, *(P*_*xx*_*–P*_*yy*_*)/2*, and *(P*_*yy*_*–P*_*zz*_*)/2*. We adopt empirical fits to the integrals of the autocorrelation function. Both *D*_*i*_ and *η* have been fit to the function in the form of ${\text{A}}\left[ {1 - exp\left( { - t/\tau } \right)} \right]$, where *A* and *τ* are free parameters. The fractional statistical error in calculating a correlation function C for molecular dynamics trajectories^23^ can be given by

4 $$ \frac{\Delta C}{C} = \sqrt {\frac{2\tau }{{T_{traj} }}} , $$

where *T*_*traj*_ is the length of the trajectory and *τ* is the correlation time of the function. In the present paper, we generally fitted over a time interval of [0,4τ–5τ].

The pair correlation function (PCF) *g(r)* or radial distribution function is usually used to describe quantitatively the internal structure of fluids.

5 $$ g\left( r \right) = \frac{V}{{4\pi r^{2} N^{2} }}\left( {\sum\limits_{i} {\sum\limits_{j \ne i} {\delta \left( {r - r_{ij} } \right)} } } \right) $$

where r is radius distance, r_ij_ and N is atom number. The PCF quantifies how the particle of interest is surrounded by other particles.

### Calculation details

Ab initio molecular dynamics were performed using the Vienna ab initio simulation package (VASP)^24,25^. The ion–electron interaction was represented by the projector augmented wave (PAW)^26,27^. The generalized gradient approximation with Perdew, Burke, and Ernzerhof corrections was employed^28^. The electronic states were populated following the Fermi–Dirac distribution^29^. For the convergence of transport property values, the size of the system is checked. Herein, 128 atoms are selected as the cell. Ni atoms were randomly distributed in the cell, with 12 Ni atoms and others are Fe atoms, and the corresponding atom ratio are 9.375 at.%. Plane wave cutoff is 400 eV enough to make sure that the pressure is converged within 1% accuracy. Time dependent mean square displacement was checked to make sure that the system is in a liquid state. The selected time step is 1 fs in all the calculations. To get the convergent transport coefficients, the total time is more than 20 ps with the beginning 2 ps discarded for equilibration. The core-mantle boundary (CMB) pressure is 136 GPa, inner-core boundary (ICB) pressure is 330 GPa, and pressures under outer core conditions were selected, such as 150 GPa, 200 GPa, 250 GPa, 300 GPa. The analytical expressions of the pressure and temperature profiles along the adiabatic curve were given by following Labrosses^30^.

It is assumed that the Earth’s outer core is in a well-mixed state with an adiabatic pressure–temperature profiles. The temperature and pressure profiles is the same as reported in^30^. The pressure *P* at radius (*r*) is

6 $$ P\left( r \right) = P_{c} + \frac{{4\pi G\rho_{cen}^{2} }}{3}\left[ {\left( {\frac{{3r^{2} }}{10} - \frac{{L^{2} }}{5}} \right)\exp \left( { - \frac{{r^{2} }}{{L^{2} }}} \right)} \right]_{r}^{{r_{c} }} $$

where *P*_*c*_ and *ρ*_*cen*_ are the pressure and density at the center of Earth, respectively; *G* is the Gravitational constant; *L* is the lengthscale; and *r*_*c*_ is the radius at the CMB. *G* = 6.6873 × 10^−11^ m^3^kg^−1^ s^−2^, *L* = 7272 km, and *r*_*c*_ = 3480 km. With the *P(r*_*c*_*)* = 136 GPa and *P(r*_*i*_*)* = 330 GPa, the pressures *P(r)* in the outer core are collected.

The adiabatic temperature *T*_*a*_ at radius *r* is

7 $$ T_{a} \left( r \right) = T_{cen} \exp \left( { - \frac{{r^{2} }}{{D_{L}^{2} }}} \right) $$

where *T*_*cen*_ is the temperature at the center of the Earth, and *D* is the lengthscale. *T*_*cen*_ = 5726 K, and *D*_*L*_ = 6203 km.

## Results and discussion

### Ab initio calculated results

**Figure 1:**
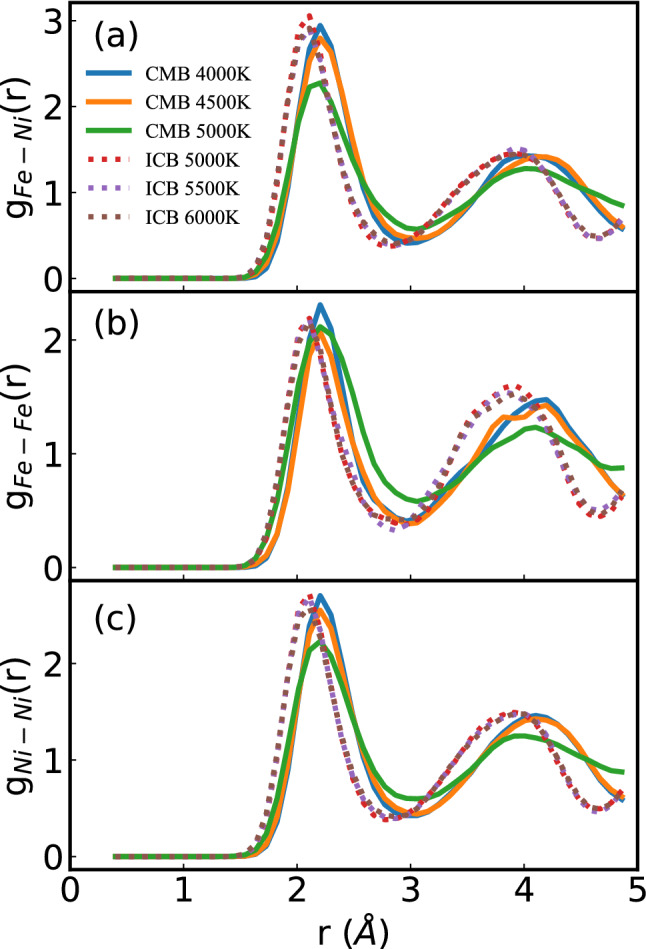
Pair correlation functions of (**a**) Fe–Ni (*g*_*Fe-Ni*_*(r)*), (**b**) Fe–Fe (*g*_*Fe-Fe*_*(r)*) and (**c**) Ni–Ni (g_Ni-Ni_(r)) in FeNi fluid under Earth’s core condition. The label ‘ICB’ and ‘CMB’ represent pressure at 330 GPa and 136 GPa, respectively.

**Figure 2:**
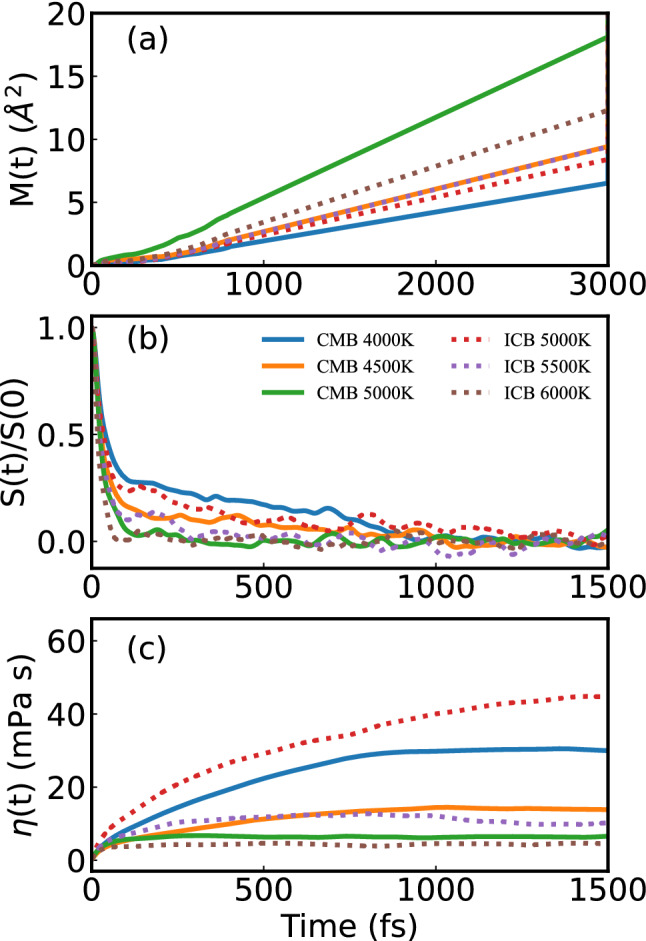
(**a**) Mean squared displacements of Fe–Fe M(t), (**b**) autocorrelation function of traceless stress tensor S(t) and (**c**) time evoluted viscosity η(t) in FeNi fluid under Earth's core condition. The label ‘ICB’ and ‘CMB’ represent 330 GPa and 136 GPa, respectively. The S(0) is velocity-velocity autocorrelation function at t = 0.

After getting the equilibrium information of the QMD, the pair correlation function *g(t)* (Fig. 1), mean-square displacement *M(t)* (Fig. 2a), and autocorrelation function of the off-diagonal component of the stress tensor *S(t)* (Fig. 2b) can be calculated. The shape of *g(r)* and *M(t)* of FeNi fluid is similar to early reported results^13^, and the calculated FeNi fluid is a close-packed liquid. The g(r) shapes of Fe–Ni, Fe–Fe, and Ni–Ni are similar, which implied that Fe and Ni atoms are similarly distributed. The *g(r)* tends towards a uniform value of 1 for a large value of r. The first peak location of *g(r)* at ICB is closer to the zero point than at CMB. From the QMD results, the equilibrium volume of Fe–Ni fluid is 8.274 Å^3^/atom at CMB and 4000 K and 6.933 Å^3^/atom at ICB and 5500 K, respectively. The *g(r)* results are consistent with the QMD calculated equilibrium volume. The peaks of *g(r)* decrease with temperature increase, especially at CMB conditions, which indicated that *g(r)* at high temperature easily converged to unity.

The mean-square displacement *M(t)* of Fe and Ni becomes a linear function of time after sufficiently large random steps (Fig. 2a). As the mobility of Fe and Ni are similar, only *M(t)* of Fe is plotted in Fig. 2a. The diffusive regime (~ t^1^) gives a self-diffusion coefficient which starts after a ballistic period of dozens of fs. At 136 GPa or 330 GPa, the *M(t)* increases nonlinearly with temperature increase. The self-diffusion coefficient is the slope of *M(t)*, it confirmed that the self-diffusion coefficient increases with the increase of temperature. The shape of *S(t),* and *η(t)* is the same as reported^31^ (Fig. 2b–c). The *S(t)* goes to zero as time tends to infinity. The present ACF at CMB in Fig. 2 looks to decay slower than that reported data at CMB in Ref.^8^. Not only the temperature and pressure states are different from^8^, but also the thermostats in our calculation are different. The ACF convergence time and behavior may be different from previous work. The error is estimated with the analytic expression in Eq.(1). At 136 GPa or 330 GPa, the *η(t → 0)* decreases with the increase of temperature. Then, the viscosity of FeNi fluid decreases with the temperature.

### Self-diffusion coefficient

**Figure 3:**
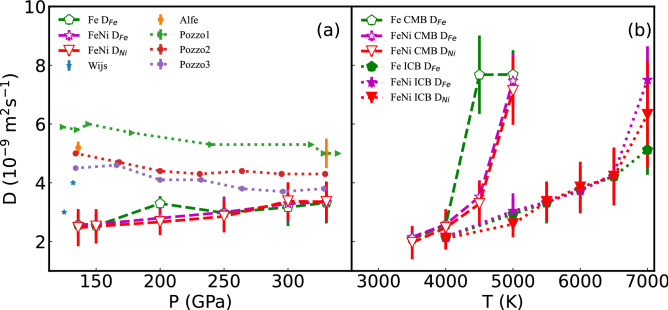
The self-diffusion coefficients (*D*_*Fe*_ and *D*_*Ni*_) of Fe and FeNi fluid under Earth's core conditions. (**a**) is along the core adiabatic, and (**b**) is temperature variation at CMB and ICB. ‘Wijs’^18^, ‘Alfe’^8^, ‘Pozzo1′^9^, ‘Pozzo2′^9^ and ‘Pozzo3′^9^ represent the self-diffusion coefficient of Fe in Fe, Fe_0.82_Si_0.10_O_0.08_ and Fe_0.79_Si_0.08_O_0.13_ fluids.

Our QMD calculations on the self-diffusion coefficient of pure Fe liquid are lower than the early reported QMD results^8,9,18^ (Fig. 3a). The main difference is that the adiabatic temperatures in this calculation are lower than the reported results. From Fig. 3b, the temperature effect on the self-diffusion coefficient exhibit Arrhenius behaviors, and self-diffusion coefficients of Fe and Ni increase with the increase of temperature. Then, it is reasonable that the self-diffusion coefficient of Fe in this manuscript is a bit lower than reported in Refs.^8,9,18^. The self-diffusion coefficients of Fe and Ni are comparable, in the orders of 10^−9^ m^2^s^−1^. Our calculated self-diffusion coefficients of Fe are consistent with the ab initio calculated results of pure Fe^8^, Fe–Si–O fluid^9^, and Fe–O fluid^11^.

Self-diffusion coefficients of Fe along the core adiabatic are in the range of [2.58,3.35] × 10^−9^ m^2^s^−1^. Self-diffusion coefficients of Ni along the core adiabatic are in the range of [2.47, 3.37] × 10^−9^ m^2^s^−1^. The self-diffusion coefficient of Fe–Ni fluid along the core adiabatic is almost constant (Fig. 3a and Table 1). The self-diffusion of Ni is a little smaller than that of Fe at the same pressure and temperature condition. From one sight, the atom mass of Ni is a little higher than that of Fe, which shows that the self-diffusion coefficient of Ni and Fe are similar, but Ni atoms move slower than Fe atoms. Considering the pressure and temperature effect (Fig. 3b), the self-diffusion coefficient is higher when at the same pressure with a higher temperature or the same temperature with lower pressure. The temperature and pressure effect on the self-diffusion coefficient in FeNi fluid can also be obtained by analyzing the of mean squared displacement (Fig. 2). Self-diffusion coefficient of Fe at CMB ranges from (2.15 ± 0.26)  × 10^−9^ at 3500 K to (7.47 ± 0.94) × 10^−9^ m^2^s^−1^ at 5000 K. The self-diffusion coefficient of Ni at CMB ranges from (1.96 ± 0.57)  × 10^−9^ at 3500 K to (7.14 ± 1.16)  × 10^−9^ at 5000 K.

**Table 1:** Self-diffusion coefficient and viscosity of FeNi fluid along the core adiabatic curve.

Pressure (P, GPa)	Temperature (T, K)	Equlibrium volume per atom (Å^3^)	Self-diffusion coefficient of Fe (D_Fe_, 10^−9^ m^2^s^−1^)	Standard error of D_Fe_ (δD_Fe_ 10^−9^ m^2^s^−1^)	Self-diffusion coefficient of Ni (D_Ni_, 10^−9^ m^2^s^−1^)	Standard error of D_Ni_ (δD_Ni_ 10^−9^ m^2^s^−1^)	Viscosity (η, mPa s)	Standard error of η (δη, mPa s)
136	3944	8.27	2.58	0.38	2.47	0.63	29.95	5.50
150	4057	8.14	2.59	0.41	2.52	0.59	23.43	5.35
200	4460	7.70	2.80	0.53	2.67	0.44	26.93	4.32
250	4863	7.36	3.00	0.52	2.85	0.54	8.55	1.44
300	5265	7.08	3.29	0.61	3.37	0.64	7.88	0.73
330	5507	6.93	3.35	0.67	3.35	0.69	12.09	1.34

### Shear viscosity

**Figure 4:**
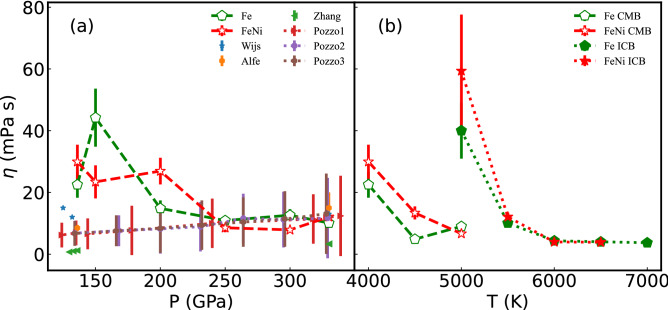
Viscosity of the pure Fe and FeNi fluid under Earth's core condition. (**a**) is viscosity along the adiabatic curve and (**b**) is viscosity under inner-core (136 GPa) and core-mantle (330 GPa) boundary pressure with different temperatures. The Fe and FeNi labels correspond to pure Fe and FeNi fluid results, respectively.

The viscosity of FeNi fluid along the adiabatic curve is in the range of [7.78, 29.95] mPa s, and the viscosity of Fe fluid is in the range of [10.10, 22.51] mPa s (shown in Fig. 4a). The viscosity of Fe-10%Ni fluid is higher than pure Fe, under ICB, and CMB (Fig. 4b). The QMD result is different from the early reported MD results^17^, which point out Ni has a negligible effect on bulk viscosity of liquid iron, about ~ mPa s^17^. Furthermore, the QMD results of viscosity are far lower than the values inferred from seismic and other measurements^13,14^, and higher than those calculated by MD^17^. The viscosity near the CMB condition reported in this manuscript is higher than the reported results by Pozzo at the same condition (Fig. 4a). The adiabatic temperature in this calculation is lower than Ref.^9^. At 136 GPa or 330 GPa, the viscosity decreases as the increase of temperature (Fig. 4b). Temperature effect on shear viscosity exhibit Arrhenius behaviors. Then, a slightly low temperature also corresponds to a high viscosity at the same pressure, and the temperature effect is obvious when at a slightly low temperature.

## Conclusion

Ab initio molecular dynamics estimates self-diffusion coefficient and viscosity of outer core are important for several purposes. The pair correlation function, mean square displacement, and autocorrelation function of traceless stress tensor are analyzed by the ab initio calculated equilibrium information. Self-diffusion coefficients of Ni along the core adiabatic range from 2.47 × 10^−9^ to 3.37 × 10^−9^ m^2^s^−1^. The viscosity of FeNi fluid along the core adiabatic range from 7.88 to 29.95 mPa s. At the core-mantle boundary, the diffusion coefficient increases with temperature increase, while viscosity decrease with temperature increase as expected.

## Data Availability

The data that support the findings of this study are available from the corresponding authors upon reasonable request.
